# Frequency and Risk Factors of Acute Kidney Injury During Diabetic Ketoacidosis in Children and Association With Neurocognitive Outcomes

**DOI:** 10.1001/jamanetworkopen.2020.25481

**Published:** 2020-12-04

**Authors:** Sage R. Myers, Nicole S. Glaser, Jennifer L. Trainor, Lise E. Nigrovic, Aris Garro, Leah Tzimenatos, Kimberly S. Quayle, Maria Y. Kwok, Arleta Rewers, Michael J. Stoner, Jeff E. Schunk, Julie K. McManemy, Kathleen M. Brown, Andrew D. DePiero, Cody S. Olsen, T. Charles Casper, Simona Ghetti, Nathan Kuppermann

**Affiliations:** 1 Division of Emergency Medicine, Children’s Hospital of Philadelphia, Philadelphia, Pennsylvania; 2 Department of Pediatrics, Perelman School of Medicine, University of Pennsylvania, Philadelphia; 3 Department of Pediatrics, University of California, Davis School of Medicine, Sacramento; 4 Division of Emergency Medicine, Ann and Robert H. Lurie Children’s Hospital, Chicago, Illinois; 5 Department of Pediatrics, Northwestern University Feinberg School of Medicine, Chicago, Illinois; 6 Division of Emergency Medicine, Boston Children’s Hospital, Boston, Massachusetts; 7 Department of Pediatrics, Harvard Medical School, Boston, Massachusetts; 8 Department of Emergency Medicine, Rhode Island Hospital, Providence; 9 Department of Pediatrics, Warren Alpert Medical School, Brown University, Providence, Rhode Island; 10 Department of Emergency Medicine, University of California, Davis School of Medicine, Sacramento; 11 Division of Emergency Medicine, St Louis Children’s Hospital, St Louis, Missouri; 12 Department of Pediatrics, Washington University School of Medicine in St Louis, St Louis, Missouri; 13 Division of Emergency Medicine, NewYork-Presbyterian Morgan Stanley Children’s Hospital, New York; 14 Department of Pediatrics, Columbia University College of Physicians and Surgeons, New York, New York; 15 Division of Emergency Medicine, Colorado Children’s Hospital, Denver; 16 Department of Pediatrics, University of Colorado–Denver School of Medicine, Aurora; 17 Division of Emergency Medicine, Nationwide Children’s Hospital, Columbus, Ohio; 18 Department of Pediatrics, The Ohio State University College of Medicine, Columbus; 19 Department of Pediatrics, University of Utah School of Medicine, Salt Lake City; 20 Division of Emergency Medicine, Texas Children’s Hospital, Houston; 21 Department of Pediatrics, Baylor College of Medicine, Houston, Texas; 22 Division of Emergency Medicine, Children’s National Medical Center, Washington, District of Columbia; 23 Department of Pediatrics, George Washington School of Medicine and Health Sciences, Washington, District of Columbia; 24 Division of Emergency Medicine, Nemours/Alfred I. duPont Hospital for Children, Wilmington, Delaware; 25 Department of Pediatrics, Sidney Kimmel Medical College at Thomas Jefferson University, Philadelphia, Pennsylvania; 26 Department of Psychology, UC Davis Health, University of California School of Medicine, Sacramento

## Abstract

**Importance:**

Acute kidney injury (AKI) occurs commonly during diabetic ketoacidosis (DKA) in children, but the underlying mechanisms and associations are unclear.

**Objective:**

To investigate risk factors for AKI and its association with neurocognitive outcomes in pediatric DKA.

**Design, Setting, and Participants:**

This cohort study was a secondary analysis of data from the Pediatric Emergency Care Applied Research Network Fluid Therapies Under Investigation in DKA Study, a prospective, multicenter, randomized clinical trial comparing fluid protocols for pediatric DKA in 13 US hospitals. Included DKA episodes occurred among children age younger than 18 years with blood glucose 300 mg/dL or greater and venous pH less than 7.25 or serum bicarbonate level less than 15 mEq/L.

**Exposures:**

DKA requiring intravenous insulin therapy.

**Main Outcomes and Measures:**

AKI occurrence and stage were assessed using serum creatinine measurements using Kidney Disease: Improving Global Outcomes criteria. DKA episodes with and without AKI were compared using univariable and multivariable methods, exploring associated factors.

**Results:**

Among 1359 DKA episodes (mean [SD] patient age, 11.6 [4.1] years; 727 [53.5%] girls; 651 patients [47.9%] with new-onset diabetes), AKI occurred in 584 episodes (43%; 95% CI, 40%-46%). A total of 252 AKI events (43%; 95% CI, 39%-47%) were stage 2 or 3. Multivariable analyses identified older age (adjusted odds ratio [AOR] per 1 year, 1.05; 95% CI, 1.00-1.09; *P* = .03), higher initial serum urea nitrogen (AOR per 1 mg/dL increase, 1.14; 95% CI, 1.11-1.18; *P* < .001), higher heart rate (AOR for 1-SD increase in *z*-score, 1.20; 95% CI, 1.09-1.32; *P* < .001), higher glucose-corrected sodium (AOR per 1 mEq/L increase, 1.03; 95% CI, 1.00-1.06; *P* = .001) and glucose concentrations (AOR per 100 mg/dL increase, 1.19; 95% CI, 1.07-1.32; *P* = .001), and lower pH (AOR per 0.1 increase, 0.63; 95% CI, 0.51-0.78; *P* < .001) as variables associated with AKI. Children with AKI, compared with those without, had lower scores on tests of short-term memory during DKA (mean [SD] digit span recall: 6.8 [2.4] vs 7.6 [2.2]; *P* = .02) and lower mean (SD) IQ scores 3 to 6 months after recovery from DKA (100.0 [12.2] vs 103.5 [13.2]; *P* = .005). Differences persisted after adjusting for DKA severity and demographic factors, including socioeconomic status.

**Conclusions and Relevance:**

These findings suggest that AKI may occur more frequently in children with greater acidosis and circulatory volume depletion during DKA and may be part of a pattern of multiple organ injury involving the kidneys and brain.

## Key Points

**Question:**

What are the mechanisms, risk factors, and outcomes associated with acute kidney injury (AKI) during pediatric diabetic ketoacidosis (DKA)?

**Findings:**

In this cohort study using data from 1359 DKA episodes in a large, multicenter, prospective study of fluid treatment during DKA, AKI occurred in 43% of episodes and was associated with greater acidosis and greater circulatory volume depletion. Children who had AKI were more likely to have subtle cognitive impairment during DKA and lower IQ at longer-term follow-up.

**Meaning:**

These findings suggest that AKI is frequent in pediatric DKA and there is a pattern of multiorgan dysfunction during childhood DKA with the possibility of common pathophysiologic mechanisms.

## Teaser

This cohort study examines the mechanisms, risk factors, and outcomes associated with acute kidney injury during pediatric diabetic ketoacidosis.

## Introduction

Recent studies have shown that the incidence of organ injuries in children with diabetic ketoacidosis (DKA) is substantially higher than previously appreciated.^1,2,3,4^ Subtle cerebral injuries occur commonly in children with DKA, which result in long-term cognitive alterations.^1,2,3,4^ Although the mechanisms of cerebral injury in DKA are under investigation, evidence supports a role for hypoperfusion and reperfusion injury as well as cerebral inflammation associated with DKA in children.^5,6,7,8,9^

One recent study demonstrated that acute kidney injury (AKI) commonly occurs in children with DKA. In a 2017 retrospective cohort study of 165 children with DKA,^10^ 64% developed AKI. In that study,^10^ AKI was reported to be more common in children with low serum bicarbonate levels, tachycardia, and hypernatremia, suggesting that severe dehydration and acidosis may play roles in triggering kidney injury. These risk factors are similar to those associated with cerebral injury during DKA,^11,12,13^ raising the question of whether factors associated with DKA severity are independently associated with increased risk of injury to multiple organs, or whether kidney and cerebral injuries during DKA might involve a single pathophysiological process. Isolated cases of severe multiple organ dysfunction syndrome during DKA have been reported^14^; however, a pathophysiological connection between DKA-related kidney injury and injury to the brain or other organs has not previously been established, to our knowledge.

Long-term cognitive changes after DKA support the possibility that acute organ injury in DKA could lead to chronic organ dysfunction.^1,2,3,15^ Furthermore, in other childhood diseases, AKI is associated with increased risk of chronic kidney disease in adulthood, particularly when repeated episodes occur.^16,17^ Therefore, additional study of AKI during DKA is important to better understand how to prevent AKI during DKA and to consider possible associations with chronic diabetic kidney disease. In this cohort study, we evaluated the frequency of and factors associated with AKI using data from a large, prospective study of pediatric DKA. We hypothesized that subtle kidney injuries occur commonly in children during DKA and may be associated with cerebral injury.

## Methods

This cohort study was a secondary analysis of a multicenter prospective study comparing effects of fluid infusion rate and sodium content on neurocognitive outcomes in children with DKA, the Pediatric Emergency Care Applied Research Network (PECARN) Fluid Therapies Under Investigation in DKA (FLUID) trial.^18^ The PECARN FLUID trial involved 13 emergency departments in PECARN and was approved by the institutional review boards of participating institutions, and informed assent was obtained for all patients. Our planned secondary analysis was included in approval and assent. The PECARN FLUID trial included patients younger than 18 years with episodes of DKA (blood glucose concentration of >300 mg/dL [to convert to millimoles per liter, multiply by 0.0555] and venous pH <7.25 or serum bicarbonate concentration <15 mEq/L [to convert to millimoles per liter, multiply by 1]). We determined the presence of AKI during DKA in all episodes with height and creatinine data available. This study is reported following the Strengthening the Reporting of Observational Studies in Epidemiology (STROBE) reporting guideline.

The PECARN FLUID Trial compared 4 fluid rehydration protocols.^18^ Exclusion criteria are described elsewhere^18,19^ and included conditions unrelated to DKA that affect mental status or cognition or substantial treatment for DKA before evaluation. Mental status during DKA was assessed hourly using Glasgow Coma Scale (GCS) scores, and short-term memory (digit span recall testing) was assessed every 4 hours during waking hours. Children 3 years and older returned for neurocognitive testing 2 to 6 months after the DKA episode, as described previously.^18,19^ This assessment included evaluation of long-term memory, IQ (Wechsler Abbreviated Scale of Intelligence for children age ≥6 years, Wechsler Preschool and Primary Scale of Intelligence for children age 3-5 years), and short-term memory (digit-span recall).^18,19,20,21,22^

Episodes of AKI were defined by the Kidney Disease: Improving Global Outcomes serum creatinine criteria.^23^ Baseline creatinine values were not available for most episodes, and most previous creatinine values in medical records were measured during illnesses. We therefore used an estimated glomerular filtration rate (eGFR) of 120 mL/min/1.73 m^2^ to calculate expected baseline creatinine levels for all DKA episodes using the Schwartz estimating equation.^10,24,25,26^ An eGFR of 120 mL/min/1.73 m^2^ was chosen for consistency with other studies of pediatric AKI^10,24,25^; however, we conducted sensitivity analyses using eGFRs of 90 mL/min/1.73 m^2^ and 110 mL/min/1.73 m^2^.^10,26^ For AKI stages 1, 2, and 3, we used Kidney Disease: Improving Global Outcomes creatinine cutoffs of 1.5-, 2-, and 3-fold estimated baseline creatinine, respectively.^23^ The maximum serum creatinine measured was used to define AKI. The first serum creatinine measured prior to initiation of DKA treatment was used to determine AKI at presentation.

Percentage dehydration was defined as the percentage difference between weight at presentation and discharge. Corrected sodium was defined as the serum sodium concentration (mEq/L) plus a factor derived using blood glucose (mg/dL) measured within 30 minutes (1.6 × [(Glucose − 100) / 100]). Heart rate and blood pressure were standardized as *z*-scores,^27,28^ indicating the number of SDs a measurement differed from the mean for age and sex. Blood pressure *z*-scores were additionally adjusted for height. Concomitant medications were reviewed to identify medications with potential nephrotoxic effects (eg, nonsteroidal anti-inflammatory drugs, statins, aspirin, cyclosporin, ciprofloxacin, trimethoprim/sulfamethoxazole, acyclovir, vancomycin, daptomycin, and furosemide). Clinically apparent cerebral injury was defined by a deterioration in neurological status leading to hyperosmolar therapy or endotracheal intubation or resulting in death.^12,29^

### Statistical Analyses

The primary analyses investigated factors associated with AKI during DKA using univariable comparisons and multivariable models. In secondary analyses, we investigated factors associated with AKI in specific patient subgroups, including those with previously diagnosed diabetes and patients for whom information was available on dehydration severity. Additional secondary analyses included sensitivity analyses varying the assumptions used to estimate baseline creatinine concentrations, and analyses exploring likelihood of AKI in repetitive DKA episodes among patients enrolled in the study with 2 episodes (per protocol, patients could be enrolled in the study no more than twice; however, 2 patients were inadvertently enrolled 3 times).

We described DKA episodes with and without AKI using means and SDs for continuous characteristics and frequencies and percentages for categorical characteristics. We compared AKI to non-AKI groups using logistic regression models. Diabetes duration and previous DKA diagnoses were compared for patients with previously diagnosed diabetes. To account for delays in result reporting, AKI was considered prior to treatment if laboratory criteria were met within 2 hours of treatment initiation, if there were no results prior.

We used a multivariable logistic regression model to estimate adjusted associations of demographic, clinical, and biochemical factors with AKI. The model included covariate adjustments for age, sex, and new-onset vs previously diagnosed diabetes. Baseline laboratory measurements were included as indicators associated with DKA severity (ie, serum urea nitrogen [SUN], glucose-corrected sodium, glucose and bicarbonate concentrations, Pco_2_, pH). Baseline heart rate *z*-score was included as an indicator of intravascular volume. Blood pressure was not included because blood pressure is often paradoxically elevated in children with DKA and is not a good indicator of intravascular volume.^30^ Diabetes duration and previous DKA episodes were not included because these data apply only to patients with previously diagnosed diabetes. The final model did not include dehydration severity owing to frequent missing data. Multivariable models were used for analyses of subgroups. Associations between AKI in initial and subsequent DKA episodes were analyzed using univariable and multivariable logistic regression adjusting for age, sex, and DKA severity (assessed by baseline serum bicarbonate) at the second enrollment.

We estimated associations between AKI and neurocognitive outcomes using conditional multivariable logistic regression (by enrolling hospital) to compare GCS declines (ie, GCS <14) between AKI groups. We used a cumulative logistic model to evaluate differences in the magnitude of GCS declines between groups, adjusting for the same covariates. We tested for an association between AKI and clinically-apparent cerebral injury using a χ^2^ test. Forward and backward digit-span recall test scores at baseline and follow-up and color and spatial task memory scores were compared between AKI groups using mixed linear regression models including random effect of enrolling hospital. IQ was compared between AKI groups using a mixed linear regression model.

A significance level of .05 was used for all statistical tests. We did not adjust significance levels for multiple tests; therefore, analyses of secondary outcomes should be considered exploratory. Analyses were performed using SAS statistical software version 9.4 (SAS Institute) from July to December 2019, with verification of results occurring as recently as October 2020.

## Results

### Frequency of AKI

**Figure 1.**
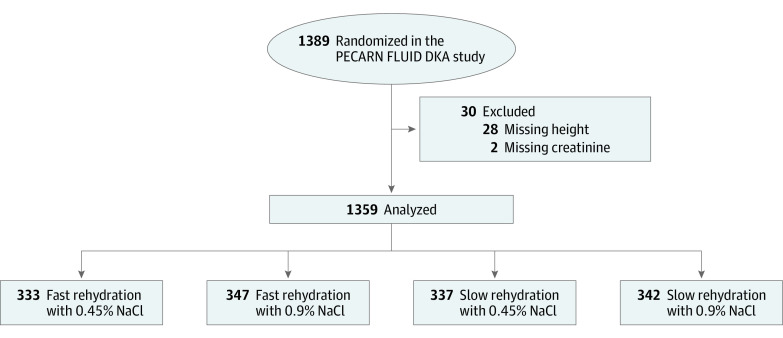
Patient Flow Diagram Patients within the parent Pediatric Emergency Care Applied Research Network Fluid Therapies Under Investigation (PECARN FLUID) diabetic ketoacidosis (DKA) study were randomized to 1 of 4 groups in a 2 × 2 factorial design including fast vs slow rehydration and 0.45% NaCl vs 0.9% NaCl rehydration fluids.

**Figure 2.**
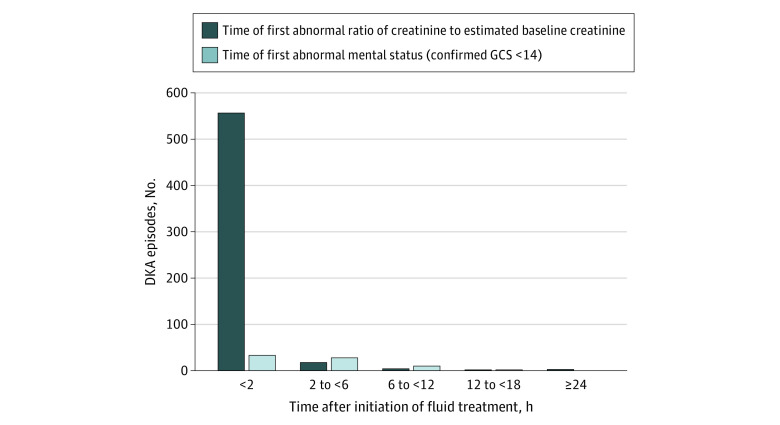
Timing of Initial Detection of Acute Kidney Injury and Impaired Mental Status The dark blue bars include data from 584 diabetic ketoacidosis (DKA) episodes with acute kidney injury. The light blue bars include data from 73 DKA episodes in which abnormal mental status (Glasgow Coma Scale [GCS] score, <14) was observed. Episodes with abnormal GCS scores at presentation and/or acute kidney injury prior to DKA treatment are included in the <2 hours bars.

Of 1389 DKA episodes in the PECARN FLUID trial, 1359 were included in this analysis. A total of 30 DKA episodes (2.2%) with missing data were excluded, including 28 missing patient height and 2 missing creatinine level (Figure 1). The mean (SD) age of participants was 11.6 (4.1) years, 727 (53.5%) were girls, and 651 (47.9%) had new onset of diabetes. AKI occurred in 584 DKA episodes (43.0%), and 553 of these episodes (94.7%) presented with AKI at diagnosis of DKA. An additional 22 episodes (3.8%) demonstrated AKI during treatment after having normal kidney function prior to treatment initiation, including 12 episodes in the fast rehydration subgroup and 10 episodes in the slow rehydration subgroup. The timing of AKI was unknown for the remaining 9 episodes (1.5%) for which first documented creatinine was more than 2 hours after treatment initiation. AKI developed 24 hours or longer after treatment initiation in 3 episodes (Figure 2). The maximum AKI stage reached was stage 1 in 332 episodes (56.8%), stage 2 in 215 episodes (36.8%), and stage 3 in 37 episodes (6.3%). In 16 episodes (2.7%), AKI stage worsened during DKA treatment, including 7 episodes in the fast rehydration subgroup and 9 episodes in the slow rehydration subgroup. No patients required dialysis.

### Factors Associated With AKI

#### Univariable Analyses

We identified multiple patient characteristics and markers of DKA severity associated with AKI in univariable analyses (Table 1). AKI was more common among children with previously diagnosed diabetes compared with those with new onset (odds ratio [OR], 2.67; 95% CI, 2.14-3.34; *P* < .001), and children with AKI were older than those without (OR per 1 year, 1.07; 95% CI, 1.04-1.10; *P* < .001). Children with AKI were more acidotic and had higher initial glucose and sodium concentrations than those without AKI, and Pco_2_ levels in children with AKI were higher than those without AKI, although respiratory rates were also higher in the AKI group (Table 1). Children with AKI also had more severe dehydration and higher initial heart rates, suggesting greater volume depletion.

**Table 1.** Patient and DKA Episode Characteristics

Characteristic	Mean (SD)^a^		OR (95% CI)^b^	*P* value
	No AKI (n = 775)	AKI (n = 584)		
Age at screening, y	11.1 (4.1)	12.2 (4.0)	1.07 (1.04-1.10)	<.001
Sex, No. (%)				
Boys	333 (43.0)	299 (51.2)	1.39 (1.12-1.73)	.003
Girls	442 (57.0)	285 (48.8)	1 [Reference]	
Previously diagnosed with diabetes, No. (%)				
No	451 (58.2)	200 (34.2)	1 [Reference]	<.001
Yes	324 (41.8)	384 (65.8)	2.67 (2.14-3.34)	
Time since onset of diabetes, y	4.7 (3.3)	5.0 (3.2)	1.02 (0.98-1.07)	.33
Previous DKA diagnoses, No. (%)				
0	73 (22.7)	112 (29.5)	1 [Reference]	.04
1-2	130 (40.5)	159 (41.8)	0.80 (0.55-1.16)	
>2	118 (36.8)	109 (28.7)	0.60 (0.41-0.89)	
Baseline laboratory values				
SUN, mg/dL	14 (5)	21 (8)	1.16 (1.14-1.19)	<.001
Serum sodium concentration (corrected for glucose), mEq/L	140 (4)	142 (6)	1.09 (1.06-1.11)	<.001
Serum bicarbonate, mEq/L	9 (3)	8 (3)	0.92 (0.89-0.95)	<.001
Pco_2_, mm Hg	26 (7)	27 (8)	1.02 (1.00-1.03)	.01
pH	7.19 (0.09)	7.13 (0.11)	0.55 (0.49-0.62)	<.001
Serum glucose concentration, mg/dL	489 (132)	565 (176)	1.41 (1.31-1.53)	<.001
Dehydration, % weight change	5.0 (3.7)	5.9 (4.1)	1.06 (1.03-1.10)	<.001
Respiratory rate at presentation	23.9 (6.0)	26.0 (7.8)	1.57 (1.33-1.86)	<.001
Heart rate, *z*-score at presentation	2.5 (1.7)	3.8 (1.6)	1.52 (1.42-1.64)	<.001
Blood pressure, *z*-score at presentation				
Systolic	1.2 (1.2)	1.3 (1.4)	1.09 (1.00-1.18)	.06
Diastolic	1.0 (1.0)	0.9 (1.1)	0.91 (0.83-1.01)	.08
Medications with possible nephrotoxic effects, No. (%)	88 (11.4)	88 (15.1)	1.39 (1.01-1.90)	.04
Before development of AKI, % with nephrotoxic meds	NA	3 (0.5)	NA	NA
After development of AKI, % with nephrotoxic meds	NA	85 (14.6)	NA	NA

Abbreviations: AKI, acute kidney injury; SUN, serum urea nitrogen; DKA, diabetic ketoacidosis; NA, not applicable; OR, odds ratio.

SI conversion factors: To convert SUN to millimoles per liter, multiply by 0.357; serum sodium concentration to millimoles per liter, multiply by 1; serum bicarbonate to millimoles per liter, multiply by 1; Pco_2_ to kilopascals, multiply by 0.133; and serum glucose concentration to millimoles per liter, multiply by 0.0555.

^a^ Missing data for previous DKA diagnoses (no AKI: 3 episodes; AKI: 4 episodes), SUN (no AKI: 59 episodes; AKI: 17 episodes), sodium (no AKI: 33 episodes; AKI: 23 episodes), bicarbonate (no AKI: 19 episodes; AKI: 11 episodes), Pco_2_ (no AKI: 36 episodes; AKI: 23 episodes), pH (no AKI: 34 episodes; AKI: 20 episodes), dehydration (no AKI: 152 episodes; AKI: 105 episodes), respiratory rate (28 episodes; AKI: 14 episodes), heart rate (no AKI: 27 episodes; AKI: 15 episodes), systolic and diastolic blood pressure (no AKI: 37 episodes; AKI: 14 episodes).

^b^ Unadjusted odds ratios compare the odds of AKI (vs No-AKI) to the reference level for categorical characteristics, a 0.1 unit change for pH, a 10-unit change in respiratory rate, a 100 unit change in serum glucose concentration, or a 1-unit change in other continuous characteristics.

#### Multivariable and Sensitivity Analyses

In multivariable analyses, older age (adjusted OR [AOR] per 1 year, 1.05; 95% CI, 1.00-1.09; *P* = .03), higher initial SUN (AOR per 1 mg/dL increase, 1.14; 95% CI, 1.11-1.18; *P* < .001), higher heart rate (AOR per 1-SD increase in *z*-score, 1.20; 95% CI, 1.09-1.32; *P* < .001), higher glucose-corrected sodium (AOR per 1 mEq/L increase, 1.03; 95% CI, 1.00-1.06; *P* = .047) and higher glucose concentrations (AOR per 100 mg/dL increase, 1.19; 95% CI, 1.07-1.32; *P* = .001), and lower pH (AOR per 0.1 increase, 0.63; 95% CI, 0.51-0.78; *P* < .001) were associated with higher risk of AKI (Table 2). Sensitivity analyses using more conservative definitions of baseline kidney function (eGFR of 90 mL/min/1.73 m^2^ or 110 mL/min/1.73 m^2^) found lower estimated AKI frequencies (90 mL/min/1.73 m^2^: 252 patients [18.5%]; 110 mL/min/1.73 m^2^: 440 patients [32.4%]); however, SUN, pH, glucose and heart rate continued to be significantly associated with AKI in all analyses (eTable 1 in the Supplement). Analyses of patients with previously diagnosed diabetes and patients for whom data on percentage dehydration (based on weight change) were available also yielded similar results (eTable 2 and eTable 3 in the Supplement). Patients reported receiving medications with possible nephrotoxic effects prior to detection of AKI in only 3 of 584 DKA episodes with AKI (all stage 1).

**Table 2.** Factors Associated With Acute Kidney Injury^a^

Variable	AOR (95% CI)	*P* value
Age at screening, per 1-y	1.05 (1.00-1.09)	.03
Sex (boys vs girls)	1.27 (0.96-1.69)	.10
Previously diagnosed with diabetes (yes vs no)	1.12 (0.79-1.60)	.53
Baseline laboratory value		
SUN, per 1 mg/dL	1.14 (1.11-1.18)	<.001
Serum sodium (corrected for glucose), per 1 mEq/L	1.03 (1.00-1.06)	.047
Bicarbonate, per 1 mEq/L	1.02 (0.94-1.11)	.62
Pco_2_, per 1 mm Hg	1.00 (0.97-1.03)	<.99
pH, per 0.1 increase	0.63 (0.51-0.78)	<.001
Serum glucose concentration, per 100 mg/dL increase	1.19 (1.07-1.32)	.001
Heart rate *z*-score at presentation, per 1-SD increase	1.20 (1.09-1.32)	<.001

Abbreviations: AOR, adjusted odds ratio; SUN, serum urea nitrogen.

^a^ Results are from a multivariable logistic regression model adjusting for each predictor in the table and no others.

### Risk of AKI With Repeated DKA Episodes

A total of 130 DKA episodes (9.6%) were repeat enrollments of patients presenting with more than 1 episode. When we compared AKI status at the first and second enrollments, the odds of AKI were 6.8-fold higher when the patient experienced AKI at the prior enrollment (OR, 6.80; 95% CI, 3.08-14.97). After adjusting for illness severity and demographic factors (ie, age, sex, and serum bicarbonate concentration at second enrollment), the AOR of AKI was even higher when the patient experienced AKI at the prior enrollment (AOR, 9.12; 95% CI, 3.65-22.79).

### Associations Between AKI and Cerebral Injury

Among 772 DKA episodes without AKI, GCS scores decreased below 14 in 14 episodes (1.8%), and of 559 DKA episodes with AKI, GCS scores decreased below 14 in 33 episodes (5.9%), but these differences were not significant in multivariable analysis (*P* = .41). However, short-term memory scores during DKA were significantly lower in patients who experienced DKA episodes with AKI compared with those who did not (mean [SD] forward digit span score, 7.6 [2.2] vs 6.8 [2.4]; *P* = .02) (Table 3). In 11 DKA episodes with AKI (1.9%) and 1 episode without AKI (0.1%), the patient developed clinically apparent cerebral injury (unadjusted *P* < .001). Additionally, patients who had AKI, compared with those without, had lower IQ scores after recovery (mean [SD score, 100.0 [12.2] vs 103.5 [13.2]; *P* = .005) (Table 3). These differences persisted after adjusting for demographic factors, including socioeconomic status, and for DKA severity. Differences in IQ were mainly noted in patients with new-onset diabetes (mean [SD] IQ score: patients with AKI, 101.4 [11.9]; patients without AKI, 106.6 [13.1]; *P* = .005) (Table 3).

**Table 3.** Associations Between AKI During DKA Treatment and Neurocognitive Outcomes

Outcome^a^	Mean (SD)		Adjusted analysis *P* value
	No AKI	AKI	
Confirmed decline in GCS score to <14, No. (%)	14 (1.8)	33 (5.9)	.41^b^
Magnitude of decline in GCS score, No. (%)			
0-1	756 (97.5)	529 (90.6)	.23^c^
2-3	12 (1.5)	30 (5.1)	
≥4	7 (0.9)	25 (4.3)	
Baseline digit-span recall test score			
Forward	7.6 (2.2)	6.8 (2.4)	.02^d^
Backward	5.8 (2.2)	5.2 (2.4)	.06^d^
IQ score	103.5 (13.2)	100.0 (12.2)	.005^e^
Among new onset patients	106.6 (13.1)	101.4 (11.9)	.005^e^
Among previously diagnosed patients	98.9 (12.1)	99.0 (12.4)	.14^e^
Memory score			
Color task	0.5 (0.2)	0.5 (0.2)	.22^d^
Spatial task	0.7 (0.2)	0.7 (0.2)	.95^d^
Follow-up digit-span recall test			
Forward	8.1 (2.2)	8.2 (2.2)	.98^d^
Backward	6.7 (2.2)	6.9 (2.3)	.92^d^

Abbreviations: AKI, acute kidney injury; DKA, diabetic ketoacidosis; GCS, Glasgow Coma Scale.

^a^ Numbers of patients with outcome data are 772 patients without AKI and 559 patients with AKI for GCS decline, 775 patients without AKI and 584 patients with AKI for magnitude of GCS decline, 696 patients without AKI and 530 patients with AKI for baseline forward digit span recall, 695 patients without AKI and 527 patients with AKI for baseline backward digit span, 501 patients without AKI and 344 patients with AKI for full scale IQ, 301 patients without AKI and 136 patients with AKI for full scale IQ among new onset patients, 200 patients without AKI and 208 patients with AKI for full scale IQ among previously diagnosed patients, 464 patients without AKI and 319 patients with AKI for item color rate, 456 patients without AKI and 320 patients with AKI for item space rate, 519 patients without AKI and 356 patients with AKI for forward digit span recall at follow-up, and 518 patients without AKI and 356 patients with AKI for backward digit span recall at follow-up.

^b^ *P* value from conditional logistic regression model adjusting for enrolling center, assigned treatment rate and sodium concentration, new onset, age, sex, and baseline SUN, Pco_2_, pH, and sodium and glucose concentrations.

^c^ *P* value from cumulative logit regression model adjusting for assigned treatment rate and sodium concentrations, new onset, age, sex, and baseline SUN, Pco_2_, pH, and sodium and glucose concentrations.

^d^ *P* value from mixed linear regression model adjusting for random effect of site and fixed effects of new onset, age, sex, socioeconomic status, and baseline SUN, Pco_2_, pH, and sodium and glucose concentrations; the effect of site was dropped from the new onset model to achieve model convergence.

^e^ *P* value from mixed linear regression model adjusting for random effect of site and fixed effects of new onset, sex, instrument, socioeconomic status, and baseline SUN, Pco_2_, pH, sodium and glucose concentrations. Site was not included in the new onset patient model to facilitate model convergence.

## Discussion

The results of this cohort study agree with previous findings that AKI is common in children with DKA and that nearly half of children with AKI had severe AKI (stage 2 or 3), suggesting intrinsic tubular injury beyond prerenal azotemia. This cohort study found that AKI was associated with greater acidosis and circulatory volume depletion, similar to associations with cerebral injury during DKA.^11,12,13^ Of note, children with AKI had higher frequencies of subtle cognitive impairment during DKA, and deficits in IQ were evident in children with AKI after DKA recovery compared with children without AKI. Importantly, IQ differences between children with and without AKI persisted after adjusting for DKA severity and demographic factors. Although the observed IQ differences were subtle, cognitive deficits associated with DKA may become greater over time.^1^ Therefore, detection of any deficits in cognition shortly after DKA recovery is of concern. Our data suggest that AKI during DKA may occur as part of a pattern of multiple organ injury involving both kidneys and brain.

The rate of AKI in our study was lower than the rate of 64.2% found in a smaller, retrospective single-center study by Hursh et al.^10^ This difference might reflect differences in cohort inclusion between the studies, particularly more severe DKA (lower mean pH values) in the retrospective group compared with our prospective cohort. Regardless, both studies confirm a high frequency of AKI during pediatric DKA.

Most patients with AKI presented with AKI prior to DKA treatment; however, 4% of patients presented with normal kidney function and developed AKI during DKA treatment, and 3% of patients had worsening of AKI during treatment. A similar frequency of progression of AKI during DKA treatment was also found in a small clinical trial by Williams et al.^31^ Similar to cerebral injury during DKA, AKI can develop as late as 12 to 24 hours after initiating DKA treatment. The development or worsening of AKI during treatment, when hydration status should be improving, suggests that some AKI in DKA may not result from prerenal mechanisms.

Our analyses indicate that AKI was associated with more severe acidosis and dehydration (higher glucose levels, heart rates, and SUN) similar to the study by Hursh et al.^10^ More importantly, we found an association between AKI and signs of cerebral injury, both during DKA treatment (alterations in short-term memory) and several months later (lower IQ scores). To our knowledge, these data are the first to demonstrate this association during DKA in children. Although occurrence of hypotension in children with DKA might explain concurrent AKI and cerebral injury, previous analyses of the study database demonstrated that hypotension occurred rarely (0.2% of patients at presentation and 3.3% of patients during DKA treatment); therefore, hypotension is unlikely to be involved.^30^ Furthermore, the association between AKI and cerebral injury persisted after adjusting for multiple aspects of DKA severity, suggesting that concurrent kidney and cerebral injury were associated with a common process and not simply reflections of a higher risk of injury to both organs in children with more severe DKA.

The cause of organ injuries in children with DKA is still poorly understood. Cerebral injury has been the most extensively studied. Although cerebral injury was previously assumed to be caused by osmotic shifts resulting from extreme hyperglycemia and rapid rehydration, recent data suggest that other mechanisms are more likely, including hypoperfusion and reperfusion injury, and damage resulting from inflammatory mechanisms.^5,6,7,8,9^ Decreased kidney perfusion likely contributes to AKI in DKA; however, evidence of cerebral inflammatory injury,^7^ and the association of AKI with cognitive dysfunction, raises the possibility of more diffuse inflammatory organ injuries, a physiological process that has not been studied in DKA, to our knowledge. Interestingly, in animal models, diabetes worsens kidney injury from ischemic events, and inflammatory mechanisms have been proposed to explain this association.^32^ Basal vacuolization of kidney tubular epithelial cells is a marker of ketoacidosis on autopsy, suggesting direct kidney injury in severe DKA.^33^ In addition, early diabetic kidney disease involves interstitial inflammation, including mononuclear cell invasion.^34^ These findings suggest a possible mechanism connecting acute and chronic kidney disease.^32,33,34^ Interestingly, among patients who enrolled in the study twice, AKI during the first DKA episode was significantly associated with the risk of occurrence of AKI during the second episode. These associations persisted after adjusting for severity of acidosis and demographic factors, suggesting either that AKI reflects specific inflammatory responses to DKA that vary among individuals or that prior episodes of AKI increase the susceptibility of the kidneys to further injury. Evaluation of factors associated with risk of AKI during DKA should be considered given these possible novel mechanisms.

### Limitations

This study has some limitations. The timing of blood sample collection varied among sites, and creatinine level frequently was not measured after acidosis resolved. Therefore, some AKI episodes may have been missed or may have occurred earlier. In addition, baseline creatinine measurements were not available for most patients and were therefore estimated. The ideal eGFR to determine baseline creatinine is controversial. We used an eGFR of 120 mL/min/1.73 m^2^ to be consistent with other pediatric AKI studies^10,24,25^; however, lower values have also been used. Sensitivity analyses using 110 mL/min/1.73 m^2^ and 90 mL/min/1.73 m^2^ yielded lower estimates of AKI frequency; however, clinical and biochemical associations were similar in all analyses. In addition, the frequency of clinically apparent cerebral injury in DKA is low and we relied on mental status and cognition as indicators of subtle cerebral injury. The extent to which subtle cognitive alterations during DKA were associated with more overt cerebral injury is unverified; however, the detection of deficits in IQ after recovery provides more convincing evidence of associations between AKI and cerebral injury. Additionally, use of potentially nephrotoxic medications prior to hospitalization was incompletely recorded; however, use of these medications in children with diabetes is uncommon. Furthermore, whether DKA influences development of chronic diabetic kidney disease is unknown. Data on urine microalbumin levels before and after DKA were not recorded, and periods longer than 2 to 6 months would likely be necessary to detect effects of DKA on chronic kidney disease.

## Conclusions

This cohort study of AKI during DKA using data from a large, multicenter, prospective study suggests new hypotheses around the development of kidney injury, and organ injuries in general, during DKA. Future investigations should focus on mechanisms associated with concurrent injury to the kidneys and brain. In addition, associations between AKI and long-term kidney dysfunction, including risk of chronic diabetic kidney disease, should be further investigated. More clearly defining the mechanisms of kidney injury resulting from DKA could have important implications for the development of new therapeutic and preventive strategies.

## Notes

Supplement. **eTable 1.** Sensitivity of Adjusted Odds Ratios of Acute Kidney Injury (AKI) to Estimation of Assumed Baseline Kidney Function **eTable 2.** Multivariable Logistic Regression Model of AKI Among Patients With Information About Dehydration Severity **eTable 3.** Multivariable Logistic Regression Model of AKI Among Patients With Previously Diagnosed Diabetes
