# Supplementary material for: Frequency and Risk Factors of Acute Kidney Injury During Diabetic Ketoacidosis in Children and Association With Neurocognitive Outcomes
Source: JAMA Netw Open. 2020 Dec 4;3(12):e2025481. doi: 10.1001/jamanetworkopen.2020.25481 (PMC7718599; doi:10.1001/jamanetworkopen.2020.25481)
Supplement: Supplement. — eTable 1. Sensitivity of Adjusted Odds Ratios of Acute Kidney Injury (AKI) to Estimation of Assumed Baseline Kidney Function eTable 2. Multivariable Logistic Regression Model of AKI Among Patients With Information About Dehydration Severity eTable 3. Multivariable Logistic Regression Model of AKI Among Patients With Previously Diagnosed Diabetes [file jamanetwopen-e2025481-s001.pdf]

Supplemental Online Content

Myers SR, Glaser NS, Trainor JL, et al; Pediatric Emergency Care Applied Research Network (PECARN) DKA FLUID Study Group. Frequency and risk factors of acute kidney injury during diabetic ketoacidosis in children and association with neurocognitive outcomes. *JAMA Netw Open*. 2020;3(12):e2025481. doi:10.1001/jamanetworkopen.2020.25481

eTable 1. Sensitivity of Adjusted Odds Ratios of Acute Kidney Injury (AKI) to Estimation of Assumed Baseline Kidney Function
eTable 2. Multivariable Logistic Regression Model of AKI Among Patients With Information About Dehydration Severity
eTable 3. Multivariable Logistic Regression Model of AKI Among Patients With Previously Diagnosed Diabetes

This supplemental material has been provided by the authors to give readers additional information about their work.

© 2020 Myers SR et al. *JAMA Network Open.*

eTable 1. Sensitivity of Adjusted Odds Ratios of Acute Kidney Injury (AKI) to Estimation of Assumed Baseline Kidney Function*

	Assumed baseline GFR 110 mL/min/1.73m^2		Assumed baseline GFR 90 mL/min/1.73m^2	
	Adjusted Odds Ratio (95% CI)	P-value	Adjusted Odds Ratio (95% CI)	P-value
**Age at screening**	1.03 (0.99, 1.07)	0.172	1.01 (0.96, 1.07)	0.683
**Sex (male vs. female)**	1.28 (0.95, 1.71)	0.104	0.97 (0.68, 1.39)	0.883
**Previously diagnosed with diabetes (yes vs. no)**	1.03 (0.71, 1.49)	0.864	1.07 (0.68, 1.67)	0.769
**Baseline BUN**	**1.13 (1.10, 1.16)**	<.001	**1.13 (1.10, 1.17)**	<.001
**Baseline serum sodium (corrected for glucose)**	1.02 (0.99, 1.05)	0.206	0.97 (0.94, 1.00)	0.066
**Baseline bicarbonate**	0.97 (0.89, 1.06)	0.500	0.99 (0.89, 1.09)	0.837
**Baseline pCO2**	1.02 (0.99, 1.05)	0.171	1.03 (0.99, 1.06)	0.124
**Baseline pH (0.1)**	**0.66 (0.54, 0.82)**	<.001	**0.59 (0.46, 0.76)**	<.001
**Baseline serum glucose concentration (100 mg/dL)**	**1.21 (1.09, 1.35)**	<.001	**1.25 (1.11, 1.41)**	<.001
**Heart rate Z-score at presentation**	**1.15 (1.05, 1.27)**	0.004	**1.21 (1.07, 1.37)**	0.002

* Results are from multivariable logistic regression models adjusting for each predictor in the table and no others (N=1154).

© 2020 Myers SR et al. *JAMA Network Open.*

eTable 2. Multivariable Logistic Regression Model of AKI Among Patients With Information About Dehydration Severity

	**Adjusted Odds ratio (95% CI)**	**P-value**
**Age at screening**	1.04 (1.00, 1.09)	0.067
**Dehydration severity based on weight change**	1.03 (0.99, 1.08)	0.155
**Sex (male vs. female)**	1.12 (0.82, 1.54)	0.480
**Previously diagnosed with diabetes (yes vs. no)**	1.19 (0.80, 1.77)	0.399
**Baseline BUN**	**1.15 (1.11, 1.19)**	<.001
**Baseline serum sodium (corrected for glucose)**	1.02 (0.99, 1.06)	0.134
**Baseline bicarbonate**	1.02 (0.93, 1.12)	0.637
**Baseline pCO2**	0.99 (0.96, 1.03)	0.756
**Baseline pH (0.1)**	**0.64 (0.51, 0.80)**	<.001
**Baseline glucose concentration (100 mg/dL)**	**1.18 (1.04, 1.32)**	0.007
**Heart rate Z-score at presentation**	**1.19 (1.07, 1.32)**	0.001

* Results are from a multivariable logistic regression model adjusting for each predictor in the table and no others (N=924).
Logistic regression models using generalized estimating equations to account for repeated measures for patients enrolled more than once had identical results with regards to factors associated with AKI.

© 2020 Myers SR et al. *JAMA Network Open.*

eTable 3. Multivariable Logistic Regression Model of AKI Among Patients With Previously Diagnosed Diabetes

	Adjusted Odds ratio (95% CI)	P-value
**Age at screening**	1.06 (1.00, 1.13)	0.059
**Sex (male vs. female)**	1.42 (0.97, 2.07)	0.069
**1-2 Previous DKA diagnoses (vs. None)**	1.05 (0.67, 1.65)	0.317
**>2 Previous DKA diagnoses (vs. None)**	0.76 (0.47, 1.24)	
**Baseline BUN**	**1.15 (1.11, 1.20)**	<.001
**Baseline serum sodium (corrected for glucose)**	0.99 (0.95, 1.04)	0.746
**Baseline bicarbonate**	0.99 (0.89, 1.09)	0.785
**Baseline pCO2**	1.00 (0.96, 1.03)	0.844
**Baseline pH (0.1)**	**0.70 (0.53, 0.92)**	0.012
**Baseline glucose concentration (100 mg/dL)**	**1.22 (1.04, 1.43)**	0.013
**Heart rate Z-score at presentation**	1.10 (0.97, 1.25)	0.139

* Results are from a multivariable logistic regression model adjusting for each predictor in the table and no others (N=598).
† Dehydration was dropped from the model due to missing weight information in 140 (20%) visits.
Logistic regression models using generalized estimating equations to account for repeated measures for patients enrolled more than once had identical results with regards to factors associated with AKI.

© 2020 Myers SR et al. *JAMA Network Open.*
